# Automatic evaluation of degree of cleanliness in capsule endoscopy based on a novel CNN architecture

**DOI:** 10.1038/s41598-020-74668-8

**Published:** 2020-10-19

**Authors:** Reinier Noorda, Andrea Nevárez, Adrián Colomer, Vicente Pons Beltrán, Valery Naranjo

**Affiliations:** 1 iTEAM Research Institute, Universitat Politècnica de València, 46022 Valencia, Spain; 2 Institute for Research and Innovation in Bioengineering (i3B), Universitat Politècnica de València, 46022 Valencia, Spain; 3 Unidad de Endoscopia Digestiva, Servicio de Medicina Digestiva, Digestive Endoscopy Research Group, IIS La Fe, Hospital Universitari i Politècnic La Fe, 46026 Valencia, Spain

**Keywords:** Image processing, Machine learning, Medical imaging, Endoscopy, Computational biology and bioinformatics, Gastroenterology

## Abstract

Capsule endoscopy (CE) is a widely used, minimally invasive alternative to traditional endoscopy that allows visualisation of the entire small intestine. Patient preparation can help to obtain a cleaner intestine and thus better visibility in the resulting videos. However, studies on the most effective preparation method are conflicting due to the absence of objective, automatic cleanliness evaluation methods. In this work, we aim to provide such a method capable of presenting results on an intuitive scale, with a relatively light-weight novel convolutional neural network architecture at its core. We trained our model using 5-fold cross-validation on an extensive data set of over 50,000 image patches, collected from 35 different CE procedures, and compared it with state-of-the-art classification methods. From the patch classification results, we developed a method to automatically estimate pixel-level probabilities and deduce cleanliness evaluation scores through automatically learnt thresholds. We then validated our method in a clinical setting on 30 newly collected CE videos, comparing the resulting scores to those independently assigned by human specialists. We obtained the highest classification accuracy for the proposed method (95.23%), with significantly lower average prediction times than for the second-best method. In the validation of our method, we found acceptable agreement with two human specialists compared to interhuman agreement, showing its validity as an objective evaluation method.

## Introduction

Capsule endoscopy (CE) is a minimally invasive alternative to traditional endoscopy. Additionally, it allows for visualisation of the entire small intestine as opposed to visualisation of only the first part through enteroscopy, or only the last part through colonoscopy. During the procedure, a video of the trajectory is recorded on a device that the patient wears on a belt, which is later analysed in the hospital. However, some of the videos cannot be analysed properly due to the presence of intestinal content, such as bile, bubbles and remainders of food, which prevent a clear vision of the mucosa.

In different clinical centres around the world, different methods are used to prepare the patient in an attempt to prevent this problem and ensure that the video can be correctly analysed. Although the manufacturer of the most widely used capsule endoscope does not suggest any specific patient preparation method other than a liquid diet, it is often argued that better results are obtained if combined with certain laxatives with different tolerance levels for patients. Several studies have attempted to compare these, but there is currently no consensus due to the absence of an objective evaluation method. Human evaluation methods, such as the one used by Pons Beltrán et al.^1^, have given contradicting results, likely due to subjectivity, while computerised methods employed so far only considered global frame information, such as the dominant colour values per frame^2^, without considering texture information and locally occurring colours in each frame.

Since the introduction of capsule endoscopy, prevention of a clear vision of the mucosa due to intestinal content has interested researchers for different purposes. Depending on the purpose and on the available knowledge, data and computing power at the time, some of them aimed to detect specific types of intestinal content, e.g. bubbles, while others focused on detection of any type. While bubbles only fully occlude the mucosa when they are grouped closely together, refraction of the light that travels through bubbles that do allow to see through, distorts the image observed through it by the camera in such a way that it is equally doubtful whether a pathology is present. Additionally, there are methods that not only aimed to detect all forms of intestinal content, but also to classify each of them separately. While most methods are based on classifying entire images, some work also aimed to locate the intestinal content or determine how much of the image is covered by intestinal content. Often, such methods apply segmentation methods afterwards in an attempt to segment the area corresponding to intestinal content, despite the absence of ground truth annotations for comparison.

The earliest work on intestinal content to our knowledge, only aiming for detection of bubbles, is the work by Vilariño et al.^3^. Their method was based on placing a threshold on the response to a bank of Gabor filters detecting the specific shape of bubbles, using an unsupervised learning method to distinguish the frames containing bubbles from the others. Wang et al.^4^. recently published their work that similarly aims to detect only non-informative bubble frames based on a threshold on a filter response, but using ring shape selective filters instead. They report an improved sensitivity over Gabor filters, while achieving the same specificity on their data set. Other work that was limited to the detection of bubbles is the work by Mewes et al.^5^. They compared different descriptors based on key points detectors, testing different combinations of key points detectors and descriptors. Additionally, they extracted statistical information from selected channels of the RGB and HSV colour histograms. Their best results were obtained with the steerable filter descriptor in combination with a Hessian-affine key point detector.

Methods detecting multiple types of intestinal content did so either in multiple stages, where each stage detects a specific type of content, or detecting all types in a single stage. A method with multiple stages was proposed by Bashar et al.^6^, detecting bubbles in the first stage and bile in the second stage. For the detection of bubbles Laguerre Gauss circular harmonic function filters were used, while bile was detected using colour histograms. In both stages it used support vector machines (SVM) for classification. This method was later modified by Sun et al.^7^, who instead made use of local quantised histograms of classic colour local binary patterns (CLBP) for the detection of bubbles, while it also replaced the SVM classifiers by a linear k-nearest neighbour (KNN) classifier.

Other methods proposed the detection of intestinal content in a single stage. Khun et al.^8^ aimed to do so by extracting texture features in the form of colour wavelet decomposition and classifying those using an SVM classifier. Seguí et al.^9^ extracted only colour information in the form of colour histograms with 64 bins, which they combined with the number of keypoints detected through speeded up robust features (SURF) keypoint detection. Apart from an SVM classifier they also separately tested a neural network (NN) classifier, generally obtaining higher accuracy for SVM.

Only a few methods have attempted to directly classify annotated regions of intestinal content instead of whole images. One of these methods is the work done by Haji-Maghsoudi et al.^10^ They proposed a multi-stage approach, where the first stage is aimed at classifying entire images, while the second classifies extracted non-overlapping regions of 32 $\times$ 32 pixels. In the first stage they use morphological feature extraction in combination with fuzzy k-means, which is fed to an NN classifier. The second stage segments the image with parameters based on its classification results and then extracts the regions. After extracting statistical features from those regions, they classify them through a second NN, thus obtaining classified regions.

In our work presented here, our aim is to develop a complete method to automatically evaluate the grade of mucosa visibility in CE videos in terms of an intuitive cleanliness evaluation score, and to validate it in a clinical setting, to ultimately provide a means for medical researchers to evaluate and compare patient preparation methods for CE procedures. In this context, in our previous work we implemented two different methods of classification of our image regions^11^, in order to compare hand-crafted feature learning and fine-tuning of convolutional neural networks (CNNs). Based on the results, we decided to fully concentrate on CNNs for the core of our overall method. Although in that work we obtained high classification accuracy for some of the methods, we found them to be too slow for practical use under our circumstances. In this work, we therefore design a light-weight novel CNN architecture to use at the core of our method, instead of the heavy state-of-the-art architectures from our earlier work, without sacrificing the high accuracy we obtained there. This should allow us to efficiently automatically learn the image features that have high distinctive value between intestinal content and clean mucosa in acceptable time. To test the achievement of these aims, we compare its performance in terms of classification accuracy, prediction times and model size in parameters and storage size, with both a fine-tuned state-of-the-art CNN architecture from our previous work and another CE-focused method trained from scratch, using exactly the same data partitions in all methods.

**Figure 1:**
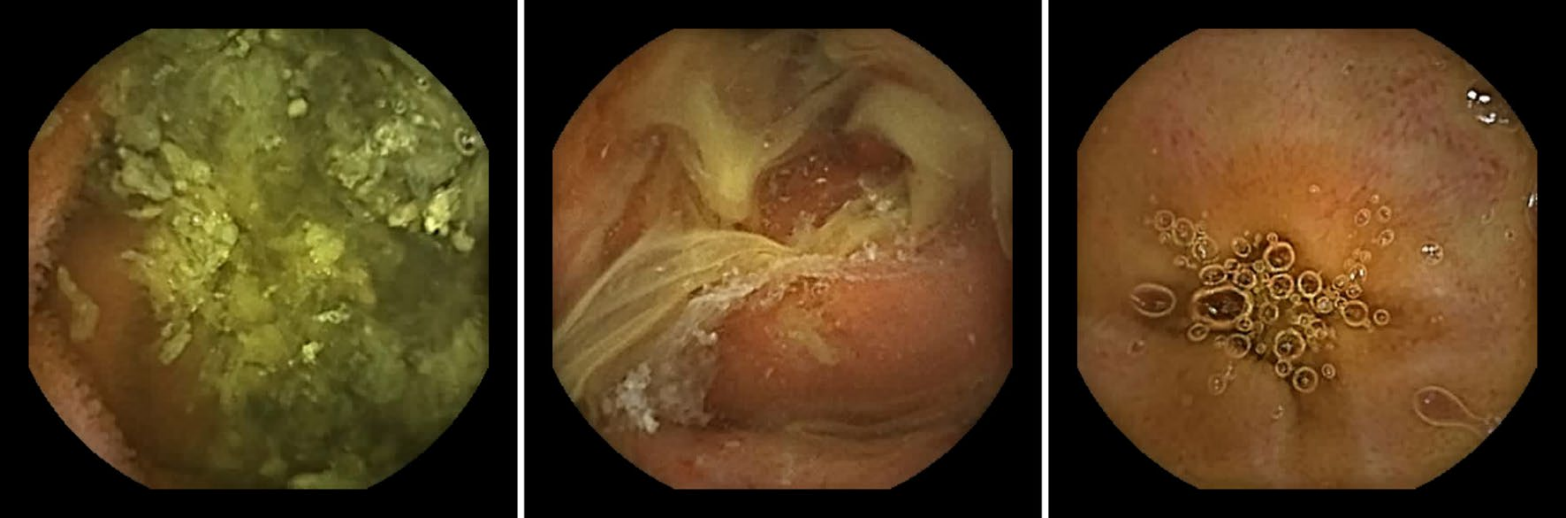
Different types of intestinal content that we aim to detect in our data set, extracted from videos of CE procedures performed with the PillCam SB 3 model, obtained as explained in “Data conditioning” section through the Rapid Reader v8.3 software, http://medtronic.com/covidien/en-us/support/software/gastrointestinal-products/rapid-reader-software-v8-3.html.

We designed three phases to design, implement and validate our method. In the first phase, we aim to create a model that is capable of automatically distinguishing between clean regions and regions containing intestinal content in CE images. Different types of such intestinal content in our data set we aim to classify are shown in Fig. 1. In the second phase, we aim to develop a method that takes a CE video as input, and estimates per-frame pixel probabilities based on the region classification results using the model trained in the previous phase. In the last phase we adapt our method to convert the pixel probabilities to evaluation scores on a cleanliness scale, which are then compared to human evaluation by two human specialists. Through an extensive procedure it is our aim to demonstrate that our method can indeed be used as an objective standard in the evaluation of visibility in CE videos and, thus, as a method of comparison of different patient preparation methods.

The remainder of this article is structured as follows. We describe our novel automatic intestinal content detection method in “Materials and methods” section, as well as the method we use to visualise the detection results on frames and the evaluation scores of mucosa visibility we extract from this. There we also describe the methods we used to validate the evaluation scores given by our method in a clinical setting. The results of our method and its validation are given in “Results” section and discussed in “Discussion” section. Finally, we present our conclusions in “Conclusion” section.

## Materials and methods

### Materials

#### Data collection

The data used in this work was collected at *Hospital Universitari i Politècnic La Fe* from Valencia, with previous approval of the study from its Ethics Committee of Clinical Research and with written informed consent from all subjects involved. The study was carried out in accordance with Spanish law for the protection of human subjects. For the training procedure of our models we collected relevant segments of 35 different videos of capsule endoscopy procedures, performed with the PillCam SB 3 model. A gastroenterologist extracted the parts corresponding to the small intestine through their official RAPID Reader v8.3 software, capable of reading the proprietary file format of the video recordings, from which we subsequently systematically extracted all frames at regular intervals of one minute. This led to a total of 563 individual frames of $576 \times 576$ pixels. For the validation in a clinical setting, we collected 30 additional videos from new capsule endoscopy procedures. From these videos we extracted frames exactly the same way, leading to a total of 854 additional frames of also $576 \times 576$ pixels for the validation procedure.

#### Data conditioning

Since we attempt not only to detect but also to locate and quantify the intestinal content, at the core of our method we use a model based on classification of regions as opposed to entire images. For this purpose, we developed an annotation tool that lets the annotator select a video from a list, after which it loads the corresponding frames that can be processed sequentially or accessed directly through another list. The tool pre-divides the area of the image that corresponds to the recording, ignoring the black frame, into patches of $64 \times 64$ pixels, with a step size of 32 pixels both in the horizontal and vertical direction, thus allowing an overlap of half in both dimensions. We verified the patch size to be adequate for our type of images and purpose in our earlier work^11^. When the annotator clicks anywhere in the image, the tool finds the centre of the closest predefined patch and marks this as dirty or clean, visualised with a red or green border around the patch respectively, depending on the mouse button used for the click. The patch can then be cleared by clicking it another time. In this way, two specialists selected those patches that either consisted completely of intestinal content (*dirty*) or were completely void of intestinal content (*clean*). We decided for such patch-based annotation because intestinal content can have unclear borders and an irregular spread, while patch-based annotation allows us to obtain annotations as detailed as desired and still allows us to capture sufficient information about its spread and transition to normal mucosa, without having to quantify the areas. Importantly, we instructed the specialists to select any encountered pathological areas naturally present in the included frames, and classify them as clean. In this way, we ended up with 26,746 clean patches and 28,547 dirty ones. Hereafter we refer to this data set as the *training set*.

For the validation of our method in a clinical setting, we aim to compare the cleanliness evaluation of our method with the independent per-image evaluation by two medical specialists. For this purpose, each of the 854 images from the 30 additional videos were annotated by two specialists with their perceived cleanliness evaluation score, as we explain in detail in “Validation in clinical setting” section. In the remainder of this article we refer to this set with its corresponding cleanliness score annotations from both specialists as the *validation set*.

#### Data partitioning

**Figure 2:**
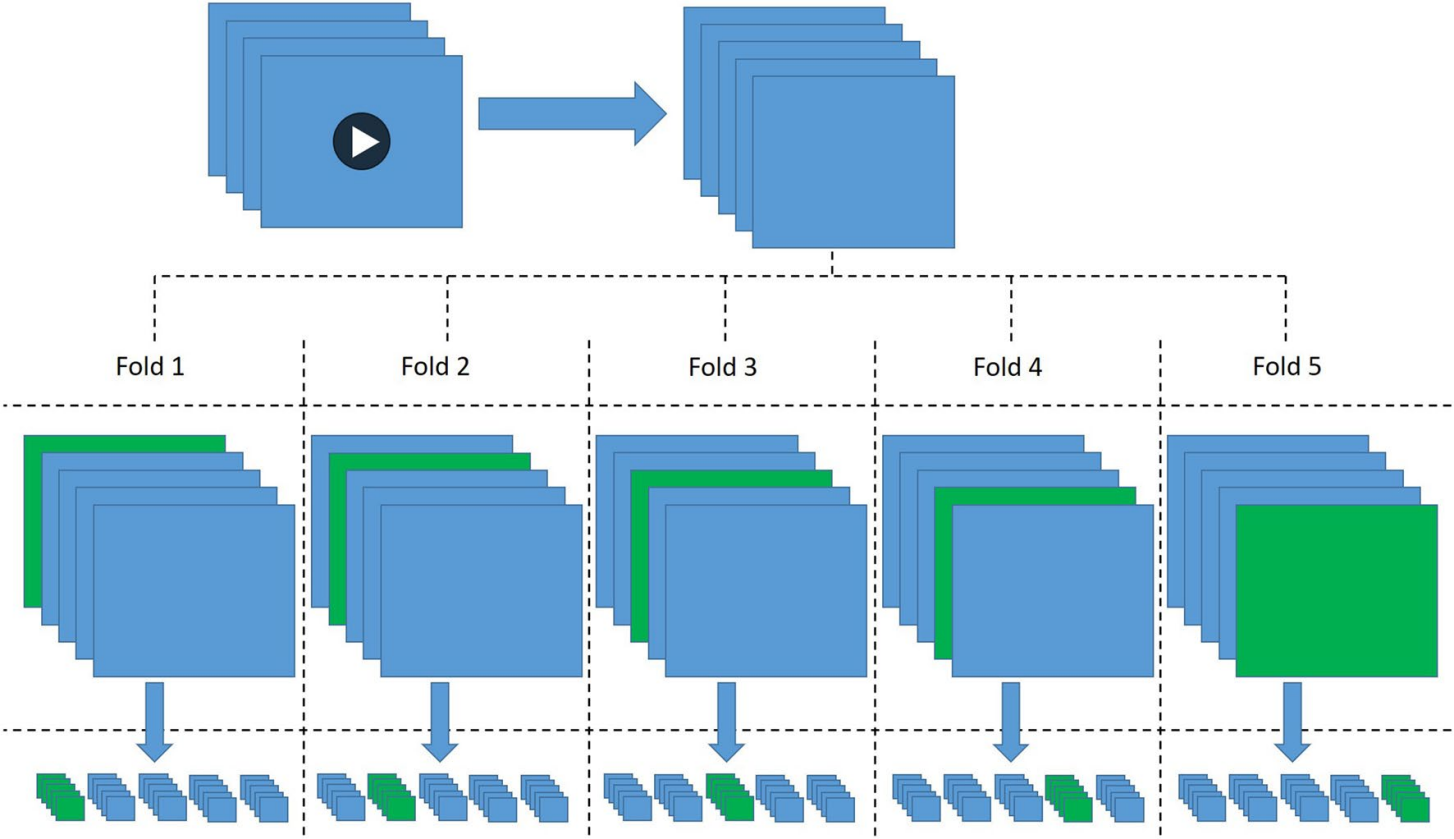
The process of our data collection and partitioning. From videos we extracted frame images in which patches were annotated by specialists. We then partitioned the images into five folds (green), while we let the remaining images be the training images in those folds. From these, we extracted the annotated patches into equivalent folds, so that we ended up with the corresponding five sets of training (blue) and test (green) patches.

To avoid overfitting to our training data and to improve the robustness of our method, we performed 5-fold cross-validation on the training set in the procedure of training of our models. Additionally, to perform fair evaluation of the performance of each model, we ensured that the patches from the test sets in these cross-validation train/test splits always originated from different images than the patches in the corresponding training sets. To achieve this, we first shuffled the entire set of images in the training set and divided them into five subsets or folds of images of equal size. For each fold, we then let the patches annotated in the corresponding images be our test patches, while the patches extracted from the images in all of the remaining folds served as our training patches in that case, thus creating five pairs of training sets and test sets of patches. This is visualised in Fig. 2, where the green images represent the test images and thus correspond to a different fold each time, while the blue images represent the images from the remaining folds. Even though our classes were relatively balanced in the original annotations, it could still happen that we end up with imbalanced data in some of our created sets due to varying number of annotated patches over the different images. Therefore, we chose to apply undersampling where necessary. Concretely, we randomly removed patches of the majority class in each of our training and test sets if the corresponding number of images was more than 10% greater than the number of of the minority class, to reduce this difference to exactly 10%. We then split each of the training sets into training and validation sets using an 80–20% split. In this way, we ended up with the final sets of patch images of the sizes given in Table 1, which we used for training and evaluation in all cases in the remainder of our method.

**Table 1:** Number of patches per fold in which we partitioned our training set for the 5-fold cross-validation in the training procedures. All models were trained and evaluated using these same partitions for training, validation and testing.

Fold	Training		Validation		Test	
	Dirty	Clean	Dirty	Clean	Dirty	Clean
1	13,579	13,902	3395	3476	4541	4823
2	13,086	14,343	3272	3586	4971	4555
3	13,063	14,310	3266	3578	5283	4809
4	13,206	14,255	3302	3564	5077	4888
5	14,018	13,667	3504	3417	3911	4162

### Methods

#### Convolutional neural networks

The core of our method aims to automatically detect regions corresponding to intestinal content in CE video frames using a convolutional neural network (CNN) architecture that we designed, implemented and trained from scratch, as opposed to transfer learning. In recent years, methods based on CNNs have shown superior performance to more traditional machine learning techniques, i.e. employing hand-crafted feature extraction in combination with specific classification algorithms, such as support vector machines, for many different purposes in biomedical imaging. Therefore, we previously compared classification of carefully designed hand-crafted features with the fine-tuning of popular CNN architectures, and found the CNNs to significantly outperform the other method^11^. Based on this experience, we decided to focus completely on CNNs for our classification method here.

We assume that the reader is familiar with the general theory of CNNs. However, here we recapture the aspects and related terms that are essential for understanding our work. It is important to consider that CNNs generally consist of two parts; the lower layers that perform the job of feature extraction, hereafter referred to as base-model, and the higher layers that together serve as classifier, hereafter referred to as top-model. The features in CNNs are mainly extracted through convolutions in convolutional layers. Where the first layers extract simple low-level features, by concatenating multiple convolutional layers, higher layers can learn to detect more complex features through non-linear combinations of the previous ones^12^. Such non-linearities can be introduced into an otherwise linear model by defining activation functions that modify the outputs of each convolutional layer. In theory, the sigmoid function appears to be a good choice, but as it is computationally expensive, alternatives have become more popular in recent years in practice and have been shown to perform equally well or even better. In our work we attempted the use of Sigmoid, Rectified Linear Unit (ReLU), Exponential Linear Unit (ELU) and Leaky ReLU. Apart from convolutional layers and their activations, the feature extraction in the base-model can be influenced by several types of non-convolutional layers that help prevent over-fitting of the model to the training samples, reduce dimensionality, introduce further non-linearities and overall improve the generalisation performance of the model. Examples of such layers are pooling layers, batch normalisation layers and drop-out layers.

CNNs are usually trained through backpropagation, i.e. optimisation of the weights of the network with respect to a chosen error function through a chosen optimisation algorithm that uses the derivatives of that function with respect to all weights and updates of their values accordingly^13^. In this work, we made use of Stochastic Gradient Descent (SGD) and Nadam, both popular choices for such optimisation algorithms. The optimisation algorithm itself and the loss function are themselves important hyperparamaters, while the optimisation algorithm on its turn depends on other hyperparameters, i.e. momentum for SGD that helps to avoid getting stuck in local minima. Other important hyperparamaters of the training procedure of CNNs are the batch size, data augmentation rates, the performance metric we choose to evaluate after each epoch to accept or reject the actualised model, the maximum number of epochs and the learning rate.

We also briefly address the later development of methods based on the CNN with Regional Features (R-CNN) method, which turned CNNs in full object detectors and rapidly became popular over the recent years. This method attempts to simplify the sliding window approach used in this work. While that approach extracts a pre-defined number of uniformly spaced regions or patches from the input image, R-CNN-based approaches often use region-proposal methods to extract regions by objectness features. Later approaches to R-CNN are regression-based, eliminating the need for region proposals and instead defining a fixed number of uniformly spaced regions of pre-defined size (also called anchor boxes) over the last feature map of the CNN network, to which it then predicts location offsets and dimension scaling factors. Although interesting, these approaches work especially well with natural objects that have clearly defined borders, while they also require bounding box annotations of each separate object. In our case, we are attempting to detect areas that consist of a mix of solids and liquids, which are sparsely defined, not uniformly shaped and have unclear borders, and are thus difficult to quantify and annotate using bounding boxes that tightly fit the region. Additionally, our approach allows us to use the known uniform overlap between our patches to create accurate detection heat maps, allowing further estimations of the degree of intestinal content as we explain later in this section.

#### Proposed architecture

In this work, we designed, implemented and trained a novel CNN architecture. We designed a new architecture to achieve high performance on our intestinal content classification, with a lower number of parameters than our previous classification methods to ensure a light-weight model with lower prediction times and a limited usage of resources. In terms of initial network depth and stride, this architecture was inspired by the architecture presented for CE images by Jia et al.^14^, for which the authors obtained a high accuracy in bleeding detection in CE images. We compared the performance of our architecture with the separately implemented base models of the architecture by Jia et al. and of the popular VGG-16 architecture^15^, all of which were trained under equal conditions, i.e. exactly the same data and partitioning of those as described above, same hyperparameters and without post- or pre-processing, to ensure a fair comparison of the classification performance of each method. In assessing the performance of our method, we specifically compared our method with VGG-16 because it performed comparably well to VGG-19 in our previous work^11^, having significantly fewer parameters. Additionally, we used the same top-model for all architectures, which is described below.

**Figure 3:**
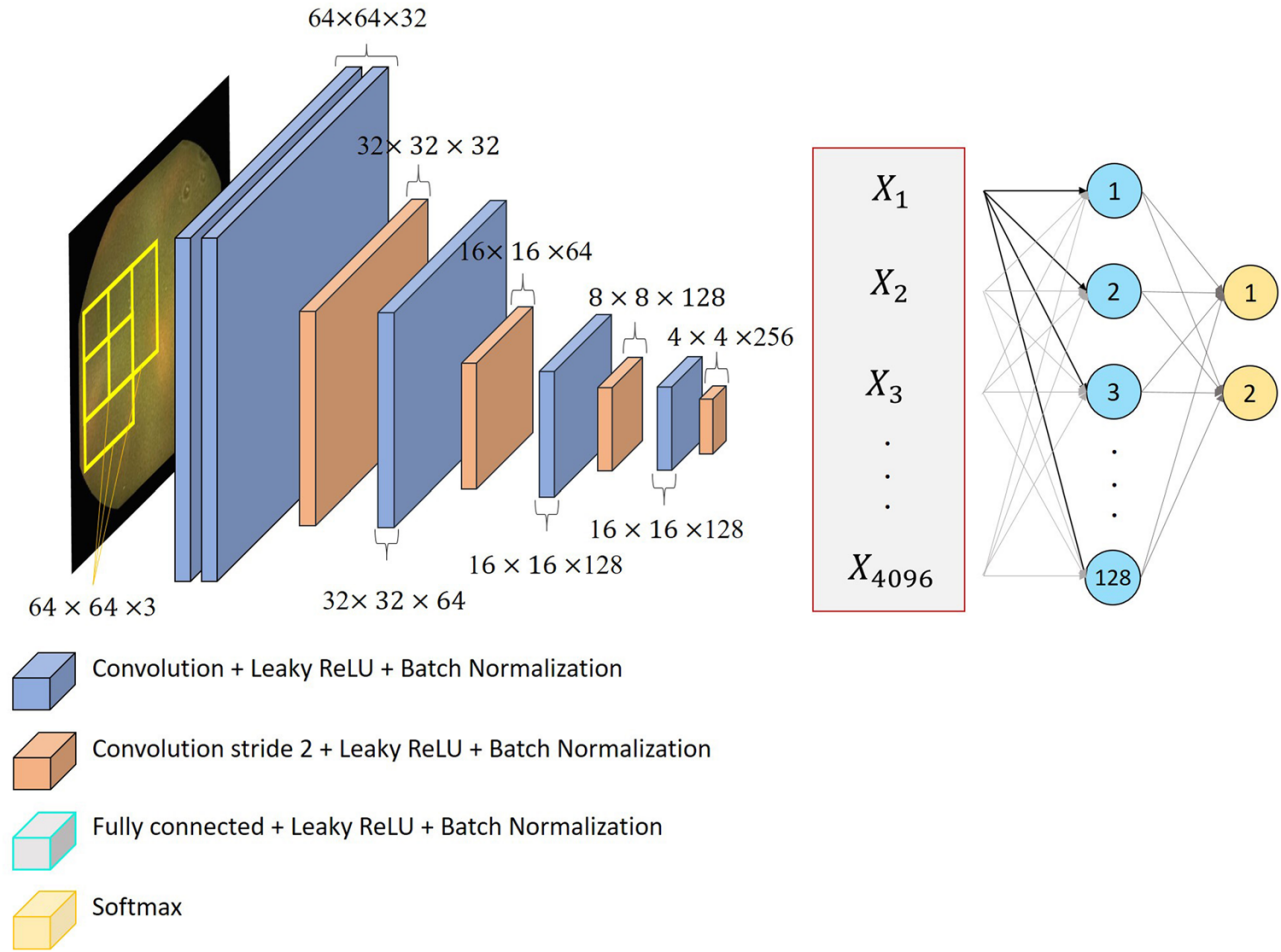
Visualisation of the CNN architecture proposed for intestinal content classification in this work.

The proposed architecture is shown in Fig. 3. The base model of this architecture consists of 4 blocks, of which the first starts with two convolutional layers with a Leaky ReLU activation function and batch normalisation, while the other blocks each have only one such layer. Each of these block ends with another convolutional layer with Leaky ReLU activation and batch normalisation, but with a stride of 2, which we verified to result in higher test classification accuracy in our case than a max pooling layer in its place, as also shown in^16^. The top model consists of flatten layers to reshape the three-dimensional output from base model of the network to a one-dimensional vector, after which two fully connected layers perform the classification. The first of these layers consists 128 neurons with a LeakyReLU activation function and batch normalisation. The last classifying layer consists of 2 neurons with the softmax activation function, resulting in outputs that correspond to the probability of the input belonging to either one of our two classes. We also experimented with the use of drop-out, but we found that it did not have any impact on the results for our data when using batch normalisation, while using drop-out instead of batch normalisation led to worse results.

#### Implementation details

We used the Keras framework^17^ for Tensorflow^18^ in Python to implement and train our architectures. We set the values of the hyperparameters for the CNN training procedure empirically. We eventually used a batch size of 16 and a learning rate of 0.0005. For VGG we used SGD as an optimiser, as it outperformed nadam, with the momentum set to 0.9 and the learning rate decay to 0. For the other models we used nadam. We did not use any data augmentation and did not rescale the input images or pre-process them in any other way; we directly used the normalised RGB colour channels of the patch images as extracted from the CE images, resulting in input vectors of size $64\times 64\times 3$ for all of our models.

Additionally, for VGG-16 we leveraged the advantage of the availability of existing weights pre-trained on the ImageNet data set to instantiate the weights. Namely, it has significantly more parameters than the other two methods due to which we cannot provide a fair comparison training it from scratch on our limited data set, which would also not be the common use case considering the average size of CE data sets. Subsequently we trained the model in two stages, adjusting the randomly initialised model head in the first stage, while we unfroze all layers and trained the entire network in the second stage.

In order to obtain a visibility evaluation score from the intestinal content detection results, we first used the overlap between patches to interpolate the detection scores at a pixel level. We did this by bilinearly interpolating the patch probabilities given by the models, assuming the given values to correspond to one of the central pixels of each patch. Concretely, we implemented Algorithm 1 to obtain the probabilities per pixel of our input image.

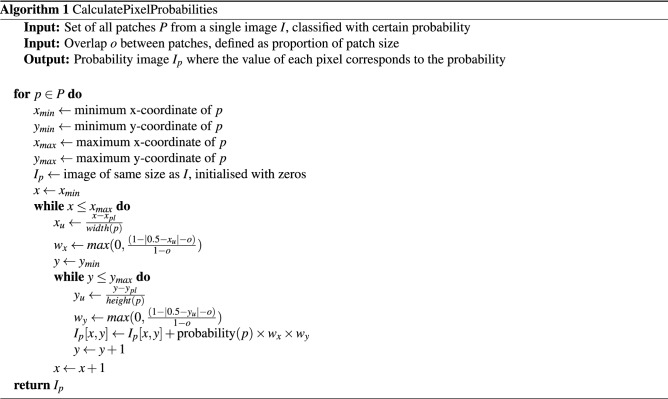



#### Validation in clinical setting

After testing the intestinal content detection performance of our method in the previous phase, in this phase our objective was to test the validity of our method in its clinical purpose, i.e. as an alternative to human evaluation in the context of cleanliness. The problem with human evaluation here, however, is that it has shown to be highly subjective as we discussed earlier, which has exactly been one of the motivation to develop our method as an objective alternative. Therefore, we do not necessarily aim for our method to adjust to any particular human cleanliness evaluation as we expect this to vary between experts (interrater reliability) and between different ratings performed by the same expert (intrarater reliability). Instead, our aim in this phase is to test whether interrater agreement between our method and human specialists is within reasonable limits of interrater agreement between human specialists only, which we discuss later in this section.

For this purpose, we had two medical experts independently evaluate each image from the validation set. They performed this evaluation independently and with a different random ordering of the images through a self-developed tool, in which for each image they had to select the category of cleanliness they perceived out of four different possibilities, which we defined as shown in Table 2. These were designed to be analogous to the categories presented in^19^ for entire videos. Hereafter we refer to the assigned category as the *cleanliness evaluation score*.

**Table 2:** Description of the different categories used to evaluate the cleanliness of CE images.

Name	Perceived level of cleanliness
Poor	Dense intestinal content impeding evaluation of the image
Fair	Substantial amount of intestinal content, allowing only partial evaluation
Good	Some intestinal content, not impeding evaluation
Excellent	No intestinal content

Similarly, we processed each of those images with our method. In our method we analogously calculated the cleanliness evaluation score by categorising the video frames into one out of four different categories, based on the average probability of a pixel corresponding to intestinal content. This categorisation is not straight-forward, as we cannot assume the described cleanliness evaluation score to be linearly correlated with the amount of intestinal content present in the image. Therefore, we needed to adjust categorisation thresholds over the probabilities of pixels being dirty according to human evaluation, instead of simply placing the thresholds on equal distance from each other in linear space. As we wanted to ensure that we could find thresholds that would generalise towards new videos on which the values were not optimised, we needed to perform this optimisation over a subset of images from the validation set, which could then logically no longer participate in the validation procedure using the found optimal thresholds. Concretely, considering our limited number of images in the validation set, we performed this learning procedure through a randomised 5-fold cross-validation procedure on the entire validation set, ensuring the frames from a single video, i.e. all images from a single patient, all ended up in the same fold. For each fold, we then validated our method using the thresholds we adjusted to the images from all of the remaining folds.

For optimising the thresholds over the remaining folds we used the evaluation scores assigned by both experts, maximising the single rater reliability of the consistency-based intraclass coefficient (ICC), denoted ICC(C, 1). The ICC is a statistic that allows two-way interrater reliability comparison involving multiple raters at once, while the single rater reliability indicates the reliability of the measurement if we are planning to use a single rater as the basis of the measurement^20^, as we plan to do with our method. The idea of using the consistency-based variant here was to reward consistency rather than absolute agreement in the optimisation, ensuring that whenever for a specific set of frames the interpretation of the amount of intestinal content differ significantly between the specialists and our method, consistency is still rewarded instead of attempting to overfit the ratings to values that contrast with the detected amount of intestinal content. At the same time, with this we aimed to reduce overfitting and thus achieve optimal generalisation towards new videos.

To evaluate the comparative ratings thus obtained, we calculated Cohen’s weighted kappa coefficient $\kappa _w$, which is a statistic measuring interrater agreement taking into account the possibility of agreement occurring by chance^21^. Apart from correction for an estimation of agreement occurring by chance as the standard kappa coefficient, this weighted version of Cohen’s kappa also allows us to merge both full and partial agreement into a single value between each two raters, along with an estimation of its 95% confidence interval. We chose to use kappa with linear weights, denoted $\kappa _1$, instead of quadratic ones, since the choice for quadratic weights tends to systematically give higher values than linear weights^22^, thus having the undesirable effect of decreasing differences between raters in our situation. The magnitude of agreement for different values of $\kappa _w$ has been reason for discussion. While some tables of the interpretation of values in different ranges have been suggested in literature, the truth is that its value also depends on other factors than agreement, such as prevalence and bias^23^, while the tendency of weighted kappa to yield consistently higher values further complicates correct interpretation of its magnitude. Therefore, here we mainly use the values for interrater comparison between the different pairs of raters without using such tables in an attempt to interpret their magnitude. In the absence of ratings from more specialists, it is also difficult to deduce any conclusions with statistical significance about the interrater agreement of our method with humans in general. Therefore, to assess whether our interrater agreement with each of the specialists individually that is within reasonable limits of interhuman agreement, we calculated $\kappa _1$ both separately over each of the different folds and over the concatenated results from all folds, in order to obtain a series of interrater agreement values that can give a more detailed picture of the agreement ranges or variances of $\kappa _1$ along with its confidence intervals over different sets of videos and overall. Finally, we calculated the single rater reliability of the two-way mixed, absolute agreement-based ICC, denoted ICC(A, 1), over the concatenated results from all folds, as $\kappa _1$ does not allow for assessment of more than two raters at the same time. This could correct for the influence of bias and prevalence $\kappa _1$ in our two-way comparisons. We calculated this statistic twice, namely once for all of our three raters and once leaving our method out, to assess the impact of our method on the resulting values and the corresponding confidence intervals.

## Results

The results we report here are threefold. First, we report the classification performance metrics on the cross-validation test sets of our training set shown in Table 1 for our own classification method, for the architecture by Jia et al.^14^ and for VGG-16. We then report the optimal thresholds we found in the threshold optimisation of cleanliness evaluation method. Finally, we report the results of the validation of our method in a clinical setting and statistically test these results.

**Figure 4:**
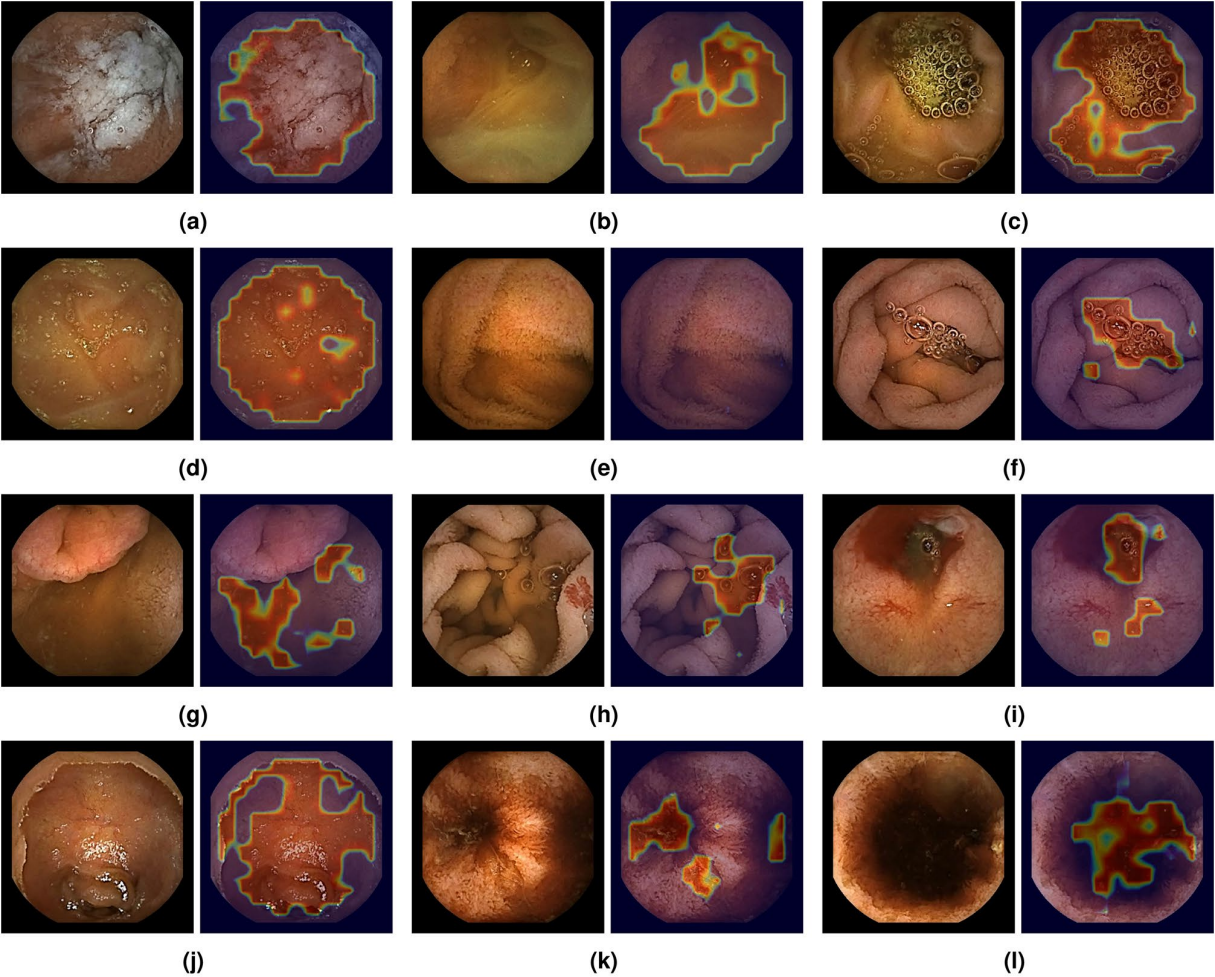
Visual intestinal content detection results of the proposed method, interpolating the probabilities per pixel from the patch probabilities and displaying as a heat map, with original images displayed next to each result. The images in (**a–f**) are selected images from our validation set showing various types of intestinal content. The images in (**g**–**i**) are selected images from the test set of our model showing intestinal content detection results in the presence of pathologies: (**g**) a polyp; (**h**) angioectasia and (**i**) an ulcer with bleeding. The images in (**j–l**) are characteristic images for which there was a significant difference between the assigned evaluation score and the evaluation scores assigned by both human specialists. Original images were extracted from videos of CE procedures performed with the PillCam SB 3 model, obtained as explained in “Data conditioning” section through the Rapid Reader v8.3 software, http://medtronic.com/covidien/en-us/support/software/gastrointestinal-products/rapid-reader-software-v8-3.html. The overlays with the detection results were created through MATLAB 2018a, http://mathworks.com/products/new_products/release2018a.html.

In our classification of dirty and clean patches, the results we obtained for each of the models trained over the different folds are given in Table 3 for all of the aforementioned methods. The proposed architecture obtains highest accuracy, followed by VGG-16 and finally by the architecture proposed by Jia et al. We also evaluated the size of each model, in terms of number of parameters and storage space, along with the average time required for a prediction of a batch of 2000 patch images, measured over the processing of 100 of such batches on an NVIDIA GeForce GTX 950 GPU using a MATLAB 2018a implementation. Here we obtained lowest values for Jia et al. with a prediction time of 0.33 s, 520,800 parameters and 4 MB of storage space on disk. This was followed by our method, with 0.50 s prediction time, 1,708,610 parameters and 20 MB storage space. Finally, VGG-16 obtained the highest values with prediction time of 0.81 s, 14,977,730 parameters and 117 MB of storage space. Eventually, integrating the model of the proposed architecture into our method of calculating the probabilities per pixel, we obtained images as shown in Fig. 4.

After training our model, we integrated it into our method to automatically evaluate the cleanliness of CE videos. In order to convert the classification of patches to an evaluation score for a frame of a CE video, we needed to determine the thresholds for the categorisation in the way we explained in “Materials and methods” section. The threshold values over the averaged detected pixel probabilities of intestinal content that we found to be optimal for assessing the videos in a way similar to human assessment, reported by their means and standard deviations over the five different folds of our validation set, were $0.42\pm 0.022$ between Excellent and Good, $0.66\pm 0.017$ between Good and Fair and $0.94\pm 0.022$ between Fair and Poor. Using these thresholds for the images in each corresponding fold, we obtained the per-fold cleanliness evaluation scores given for our method. All per-frame scores thus obtained for our model for the videos in each fold are given in Supplementary Table S1 online. All scores assigned by specialist 1 and specialist 2 are given in Supplementary Table S2 and Supplementary Table S3 online respectively, sorted in the same per-fold order for convenience.

We separately analysed the detection results on the images that contained pathologies in our test set, which were thus not used for training the corresponding model as described in “Data partitioning” section. With the help of one of our experts, we identified 10 images in this set showing clear pathologies. Within these, our expert diagnosed 2 cases of active bleedings, 3 of ulcers, 5 of angioectasia and 1 of a polyp. Through manual analysis of the intestinal content detection results with the proposed method over these images, we found that one of the ulcers was partly detected as intestinal content, while all of the remaining pathologies were correctly classified as clean intestine by our method. The case of the polyp is shown in Fig. 4g, a case of angioectasia in Fig. 4h and the case of ulcer that was partly detected as intestinal content in Fig. 4i, with the bleeding classified as clean intestine in the same image.

**Table 3:** Results for the different methods over each of the different folds, with the average for each method in the bottom row.

Method	Fold	Accuracy (%)	Sensitivity (%)	Specificity (%)	MCC
VGG-16	1	94.27	96.81	91.57	0.8861
	2	94.68	92.29	96.86	0.8939
	3	94.35	98.65	90.44	0.8906
	4	95.02	98.10	92.06	0.9023
	5	95.66	96.03	95.27	0.9132
	Average	94.80	96.38	93.24	0.8972
Jia et al.	1	93.40	97.26	89.30	0.8701
	2	93.46	92.54	94.31	0.8689
	3	93.84	97.40	90.59	0.8793
	4	93.96	96.87	91.16	0.8808
	5	94.96	96.06	93.79	0.8992
	Average	93.92	96.03	91.83	0.8797
Proposed	1	94.96	95.92	93.94	0.8992
	2	95.67	94.84	96.44	0.9134
	3	95.05	98.09	92.28	0.9028
	4	94.77	96.64	92.97	0.8961
	5	95.71	95.41	96.04	0.9143
	Average	95.23	96.18	94.33	0.9051

**Figure 5:**
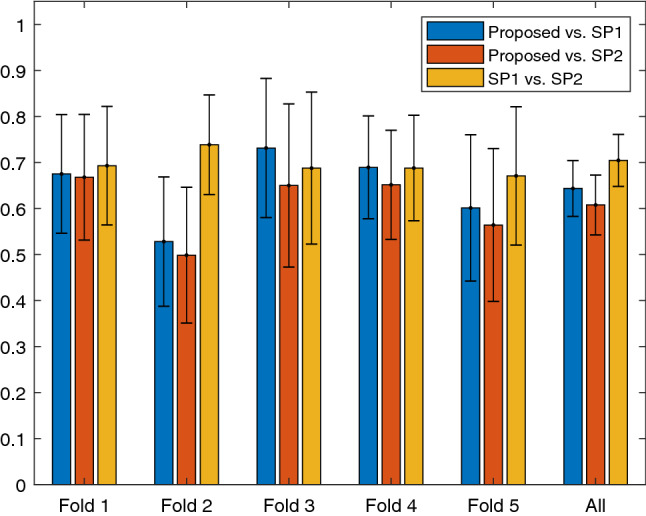
The agreement values of $\kappa _1$ measured over the different folds and over the concatenated results of all folds, each plotted with its corresponding 95% confidence interval. Figure created using MATLAB 2018a, http://mathworks.com/products/new_products/release2018a.html.

For the validation of our method, we calculated the interrater statistics that allow us to validate our method as described in “Validation in clinical setting” section. On a per-fold basis, we calculated $\kappa _1$ for each pair of different raters, allowing us to validate our method through comparison with the evaluation by human specialists. We used a MATLAB implementation of weighted kappa^24^ to perform this calculation. The resulting $\kappa _1$-values and their corresponding 95%-confidence intervals are given in Fig. 5. Except for the case of fold 2, the values found for our method were always within the 95% confidence interval of the $\kappa _1$ between the human specialists. Overall, concatenating the ratings over all folds, we measured a $\kappa _1$ of 0.643 and 0.608 for our method (Proposed) with specialist 1 (SP1) and specialist 2 (SP2) respectively, while we measured a $\kappa _1$ of 0.704 for the agreement of both specialists with each other, with confidence intervals of (0.583, 0.704), (0.543, 0.672) and (0.648, 0.761) respectively. Finally, using SPSS v25.0 we calculated ICC(A, 1) both over only the ratings of the human specialists and over all raters at once, to measure its change with the exclusion or inclusion of our method respectively. Over the two human raters we thus calculated an ICC(A, 1) of 0.817 with a confidence interval of (0.745, 0.864), while including our method among the raters ICC(A, 1) was 0.770 with a confidence interval of (0.739, 0.798).

## Discussion

While we aimed to design a CNN architecture that would approach the performance of VGG-16 with significantly fewer parameters and a significantly reduced prediction time for faster processing of videos, our results show that with the proposed architecture we can even improve classification performance over VGG-16, despite having fewer than 2 million parameters as opposed to nearly 15 million for VGG-16 and requiring only 62% of the prediction time of VGG-16. Compared with the architecture proposed by Jia et al., we obtained significantly higher accuracy for the proposed architecture, at the cost of more parameters and a 52% increased average prediction time. Additionally, through the inclusion of images that showed pathologies, our method correctly recognised bleeding, angioectasia, a polyp and most of the ulcers as a part of the clean intestine. Although this looks promising, it will be necessary to test this on a greater set of pathological images in the future before we can draw any conclusions. Of our test cases, only one ulcer appeared to be partly detected as intestinal content. For ulcers, the situation is more complicated than for the other pathologies, as they are often accompanied by intestinal content in the case of perforated ulcers, while in other cases they can still be difficult to distinguish from intestinal content in still image frames even for human experts. In the case of Fig. 4i, for example, the part of the ulcer detected as intestinal content has very similar characteristics to some of the intestinal content in Fig. 4a, while it also has bubbles in its vicinity.

In our validation procedure, we found that the agreement between our method and each of the specialists individually over all images approached the interhuman agreement, yet the values fell just outside of the interhuman 95% confidence interval. Especially in the case of fold 2 our method performed significantly worse than in other folds, scoring a lower agreement with either one of the human specialists than the lower limit of the 95% confidence interval of the $\kappa _1$ of the two specialists with each other, while in all of the other folds it was always within that interval, achieving higher agreement with SP1 for fold 3 and fold 4. Observing the case of fold 2 in greater detail, we noticed that one of the videos in this fold contained a significant number of frames on which bleeding could be observed, where our method assigned evaluation scores of excellent cleanliness, while both specialists agreed on poor cleanliness. Measuring the ICC(A, 1) both with our method included and with our method excluded, we found that the resulting value of the single rater reliability remained within the 95% confidence interval of the single rater reliability of ICC(A, 1) with our method excluded. Although we cannot prove significance as discussed earlier, we believe that this information in combination with the comparative values found for $\kappa _1$ does give a good indication that our method’s agreement with the human specialists is within reasonable limits of inter-human agreement.

It is important note that even though the specialists performed the validation procedure independently, they work together in the analysis of CE procedures on a regular basis, which may make them more prone to agreeing with each other in their perception of cleanliness than what would be the case between specialists from different institutions. We also highlight that, when we reviewed the frames with highest disagreement between our method and both human specialists, in many cases both of them no longer agreed with the evaluation score they assigned in first instance. This demonstrates the lower intrarater reliability of human evaluation due to the earlier discussed subjectivity, while intrarater reliability is always 1 in the case of our method, as it will consistently assign the exact same evaluation scores to each image regardless of the number of repetitions.

Through reviewing the mentioned frames, we identified three specific cases for which the evaluation by our method significantly differed from human evaluation. Examples of these are shown in Fig. 4j,k,l. In the first case, shown in Fig. 4j, the capsule around the camera appears to be caught in a bubble itself. As our method has been trained to detect bubbles, it may detect the smooth surface of the mucosa without its characteristic texture, while it may also detect that the light emitted by the capsule is reflected by the surface of the bubble. We argue that it is difficult for our method to distinguish between the case of the capsule being inside a greater bubble, allowing us to observe the mucosa nearly perfectly, and the case of smaller bubbles on the surface of the mucosa that reflect the light much more rigorously and thus do not allow for correct observation of the mucosa behind it. However, although the general occurrence of such frames is relatively limited, we believe that in future work we could address this issue by actively collecting more such images to include in our training set. The second type of images with a significant difference images that show bleeding, as shown in Fig. 4k, which were numerous in one of the videos in Fold 2. In these images, the blood is not detected as intestinal content by our method, which is in fact desired behaviour as the scale addresses the remainders of food, bile and intestinal liquid. However, our specialists did assign a low evaluation score in the presence of bleeding. We argue that this may be an effect of human psychology, assigning a bad score to an image in the presence of diseases despite evaluating other factors, which could show an important advantage of our method in its objectiveness. Finally, our method sometimes detects sparse intestinal content in the lumen hole where human observers do not, as shown in Fig. 4l. Even though it is not a false detection, it can be argued that, as the mucosa in the darkness cannot be observed either way, it does not impede visibility. In future work, it would be interesting to investigate how the relative location of intestinal content, e.g. against the background of the lumen hole or against the background of the near intestinal wall, influences the cleanliness in a frame as perceived by humans. The presence of intestinal content against the background of the lumen hole does, however, provide information about the preparation of the intestine that we can expect to normalise over the course of an entire video.

Considering the limited number of specialists available for the cleanliness evaluation of the images in our validation set, we were forced to use part of these images, annotated with both assigned evaluation scores, for adjusting the categorisation thresholds. This is a limitation of our method, as it could cause our method to over-adjust to the specific annotators. Ideally, we would like the scores used to adjust the thresholds of our method and the scores used to validate the method to be completely independent from each other, e.g. originating from different observers, as this would show that our thresholds generalise well to other observers. We would like to revisit this in our future work when we could obtain more data from different clinical centres around the world. We do, however, emphasise that we did guarantee independence between validation and adjustment of the thresholds in terms of our data, as we not only used different videos for each of those, but additionally obtained entirely new videos for the validation procedure, ensuring that none of the videos used for validation was also used in the training procedure.

## Conclusion

In this work, we proposed a method to automatically evaluate the cleanliness of the small bowel in videos of CE procedures for comparison purposes. In this way, we aim to provide medical researchers with a means for accurate and objective evaluation of the effect of different preparation methods for CE patients. This would allow them to reach consensus on the optimal method, which has not been possible so far due to conflicting results in studies in the absence of such a means.

The core of our proposed method is a model based on a CNN architecture we designed, capable of classifying image patches into intestinal content or clean mucosa, which was inspired by the architecture presented by Jia et al.^14^. For this model we obtained a high accuracy of 95.23%, surpassing the accuracy of the model we trained on the popular VGG-16 architecture using exactly the same data, while we achieved a significantly lower prediction time and decreased number of parameters. We also obtained significantly higher accuracy for this model than for the one based on the original architecture.

Using this model for the patch-based classification at its core, the method we proposed estimates pixel probabilities from the patch probabilities and visualises these intuitively, while it also determines the average probability of a pixel of corresponding to intestinal content and finally converts this to an evaluation score on a discrete scale with four categories. In this way, the proposed method is capable of automatically evaluating frames of CE videos on a scale that is meant to be equivalent to the cleanliness evaluation score proposed in^19^. In the validation of our method, evaluating the scores thus assigned to 854 frames extracted from 30 different CE videos using a 5-fold cross-validation procedure for threshold adjustment, we found acceptable agreement with each of the human specialists, despite strong disagreement between our method and both specialists on frames that showed bleeding in one fold in particular, where our method correctly did not detect the blood as intestinal content. In a three-way comparison, using the two-way mixed, absolute agreement-based intraclass correlation coefficient for single rater reliability, we also showed that the value found when including our method did not move outside the 95% confidence interval found for both specialists alone.

Despite promising results in the validation of our method, we have observed and discussed some current limitations of our method, most of which we believe we can overcome by adapting our method in future work as discussed previously. Namely, it may be interesting to investigate the influence of the relative location of detected intestinal content in the cleanliness as perceived by humans. Additionally, we aim to further optimise our thresholds on new, more extensive data sets using video material being currently collected from different medical centres world-wide, to assure that the thresholds are averaged out over a wide cohort of medical specialists to obtain a satisfactory agreement with each one of them individually, and to simultaneously further extend the validation of our method in a clinical setting involving a greater number of specialists from different institutions.

## Supplementary information

Supplementary material 1

Supplementary material 2

Supplementary material 3

## Data Availability

The patched labelled CE images used for training and evaluating the models can be found at: https://cvblab.synology.me/PublicDatabases/CECleanliness/CECleanlinessTraining.zip. The CE images used for the validation in a clinical setting can be found at: https://cvblab.synology.me/PublicDatabases/CECleanliness/CECleanlinessValidation.zip.
